# Risk factors for hypoactive delirium in patients with nontraumatic ARDS: a prospective observational study

**DOI:** 10.1038/s41598-024-57525-w

**Published:** 2024-03-24

**Authors:** Fuyan Lian, Fei Li, Xuemei Tang, Yuan Yuan

**Affiliations:** 1 Department of Critical Care Medicine, Gansu Provincial People’s Hospital, Lanzhou, 70030 China; 2 Department of Infection Management, Lanzhou University Second Hospital, Lanzhou, 70030 China

**Keywords:** Hypoactive delirium, Nontraumatic ARDS, Intensive care unit, Risk factor, IL-6, Diseases, Risk factors

## Abstract

To investigate the incidence, characteristics and risk factors for hypoactive delirium in patients with nontraumatic acute respiratory distress syndrome (ARDS) and to explore the independent risk factors associated with hypoactive delirium and provide new ideas for early prediction and treatment. Hypoactive delirium is a known serious complication in ARDS patients, and currently, there are no effective early detection models or clinical prediction tools, and there is a lack of clinical treatment. This study included nontraumatic ARDS patients who stayed in the intensive care unit (ICU) for more than 24 h and were older than 18 years. A total of 205 ARDS patients admitted to the ICU of Gansu Provincial People's Hospital between December 2021 and February 2023 were selected. Demographic data, clinical characteristics and laboratory test results were collected within 24 h after the patients entered the ICU. Multivariate logistic regression analysis was used to investigate risk factors, evaluate the clinical prediction effect of the model and construct a nomogram for visual display. The incidence of hypoactive delirium among the patients included in the study was 41%. Patients with hypoactive delirium had hypertension; diabetes mellitus; Acute Physiology and Chronic Health Evaluation II (APACHE II) scores ≥ 15; and increased procalcitonin, C-reactive protein (CRP), lactic dehydrogenase and interleukin-6 (IL-6) levels compared with those without hypoactive delirium. Logistic regression analysis revealed that diabetes mellitus (OR 3.305, 95% CI: 1.866–12.616; p = 0.047), CRP level (OR 1.002, 95% CI: 1.001–1.023; p = 0.044), and IL-6 level (OR 1.045, 95% CI: 1.017–1.063; p = 0.001) were independent risk factors for hypoactive delirium. After receiver operating characteristic (ROC) curve analysis, calibration plot and decision curve analysis (DCA) confirmed that the clinical prediction ability of this study model was satisfactory, and a nomogram was drawn for visual display. Hypoactive delirium is a common serious complication in nontraumatic ARDS patients. Our logistic regression model not only effectively predicts hypoactive delirium early but also reveals potential clinical therapeutic targets.

## Introduction

Delirium is a set of acute brain dysfunctions that is highly prevalent among critically ill patients and is mainly characterized by disturbed sleep, memory deficits, disorientation, disturbance of attention, abnormal emotions, changes in cognitive function and an altered level of consciousness^1^. Delirium is a potentially life-threatening disease in the ICU, affecting up to 80% of mechanically ventilated elderly patients; it is associated with increased mortality and morbidity, prolonged ICU length of stay, prolonged mechanical ventilation length, acquired cognitive impairment, acquired dementia, and increased treatment costs^2,3^.

According to the characteristics of the associated psychomotor abnormalities, delirium can be divided into three subtypes: hyperactive delirium, hypoactive delirium and mixed delirium^4^. Previous studies have shown that hyperactive delirium is a more common form of delirium outside the ICU, while hypoactive delirium and mixed delirium are more common in the ICU. Some studies have also shown a positive correlation between hypoactive delirium and disease severity^2,5,6^.

In recent years, we have observed “silent hypoxemia” in most patients with ARDS, including neurological manifestations of confusion, difficulty communicating, inability to recognize people, and lack of logical expression^7^. ARDS patients with hypoactive delirium are often considered to be sleepy or drowsy because of their age, hypoxemia, or medication use. Compared with the other two subtypes, hypoactive delirium is “quiet”; the clinical symptoms of hypoactive delirium are not obvious, and it is easily undiagnosed or misdiagnosed^8,9^. A study revealed that almost all survivors of ARDS have symptoms of delirium, and 80% of patients experience memory loss, difficulty concentrating, and decreased reaction speed even 1 year later^10^. Hypoactive delirium is associated with poorer outcomes, and one study reported that 75% of cases of hypoactive delirium were not detected by clinicians^11^. Early diagnosis of hypoactive delirium and early treatment can reduce patient mortality.

To date, there are no reliable biomarkers or rapid monitoring methods for diagnosing hypoactive delirium in critically ill ARDS patients. According to previous reports, the appropriate treatment is different depending on the delirium subtype, so early accurate diagnosis is very important^2^. In this study, we conducted a prospective, observational study to identify the independent risk factors associated with hypoactive delirium in ARDS patients. We hope our findings not only contribute to rapid diagnosis but also suggest possible clinical interventions to minimize the risk and harm of hypoactive delirium in ARDS patients.

## Materials and methods

### Patients

Using convenience sampling methods, 205 ARDS patients admitted to the ICU of Gansu Provincial People's Hospital between December 2021 and February 2023 were selected. The inclusion criteria for patients were as follows: (1) aged 18 years or older; (2) diagnosed with ARDS according to the Berlin criteria^12^; and (3) agreed to participate in the study and provided informed consent. The exclusion criteria were as follows: (1) transfer, cardiopulmonary resuscitation or death within 24 h of admission to the ICU; (2) anoxic brain injury, neuromuscular disorders, cerebrovascular disease, intracranial infection or dementia; (3) history of psychiatric disease, alcoholism or drug dependence; (4) hearing and language communication disorders; and (5) trauma-associated ARDS. This study was approved by the ethics committee of Gansu Provincial People's Hospital (No. 2022-469).

### Diagnostic criteria for delirium

All patients were assessed by well-trained doctors and/or nurse investigators. Patients were assessed at 8 AM and 6 PM within 24 h of admission, and delirium was defined as a positive CAM-ICU score at any time point^13^. All patients were first assessed using the Richmond Agitation-Sedation Scale (RASS) score, which ranges from − 5 to + 4 and represents varying degrees of sedation from coma to aggression. The presence of delirium was further assessed for patients with scores of − 3 to + 4^14^. A positive CAM-ICU score accompanying a RASS score of − 3 to 0 was judged as hypoactive delirium, and a RASS score of + 1 to + 4 was judged as hyperactive delirium. Fluctuations between hypoactivity and hyperactivity suggest mixed delirium^8,13,14^.

### Data collection

We collected data, including age, sex, medical history, and APACHE-II score. The laboratory test indicators were peripheral blood neutrophil count; lymphocyte count; and hemoglobin, albumin, brain natriuretic peptide (BNP), CRP, lactate dehydrogenase, procalcitonin, serum magnesium, serum sodium, and IL-6 levels. Laboratory tests were performed within 24 h after the patient entered the ICU, and all information was obtained from the hospital information system (HIS). The APACHE II score consists of three parts: acute physiological score, age score and chronic health score. This scoring method is mainly applied to patients in the intensive care unit (ICU) for the evaluation of physiological parameters to determine the severity of the patient’s condition. Patients with more than 15 points are classified as severe. The equipment used for laboratory tests included a SYSMEX K-4500 hematology analyzer (Japan), an Olympus AU5400 automatic biochemistry analyzer and an Abbott i-4000 automatic chemiluminescence analyzer (USA). Each step was performed according to the standard operating procedure.

### Statistical analysis

In this study, we first performed univariate and multivariate logistic regression analyses with the IBM SPSS 26.0 system to calculate odds ratios and 95% confidence intervals. The Kolmogorov–Smirnov test was used to determine the normality of the distribution of continuous variables, which are expressed as medians and interquartile ranges (25–75th percentiles), and to select indicators that were significantly different for delirium prognosis. Categorical variables are presented as 0 or 1 (0 represents no outcome event, and 1 represents an outcome event that has already occurred). Student’s t test or the Mann–Whitney U test was used to determine whether categorical variables were significant for outcome events, and p < 0.05 was considered to indicate statistical significance.

The development process for the clinical prognostic model was implemented in R (version 4.1.2). The R packages used in this study included “foreign”, “survival”, “rms”, “pROC”, “rmda” and “nricens” packages. Patients were randomly selected and assigned to the training set (70%) or the validation set (30%). The outcome was whether the patient experienced delirium after hospitalization. Three independent risk factors for delirium outcome were identified, nomograms were drawn using all patient datasets, and visual models were constructed. The performance of the prognostic model for the development of hypoactive delirium in the validation set was further estimated by the area under the curve (AUC) and bootstrap resampling methods (1000 iterations). Finally, risk assessment of the clinical prognosis model was performed by decision curve analysis (DCA). p values < 0.05 indicate statistical significance. The study was conducted according to the Declaration of Helsinki (revised 2013).

### Ethics approval and consent

All studies were performed according to the institutional guidelines of the Ethics Committee of Gansu Provincial People’s Hospital.

## Results

### General information

Among the 205 ARDS patients included in this study who met the inclusion criteria, more than half (70.2%) had pneumonia; 27 patients (13.2%) had severe pancreatitis, 24 (11.7%) had septic shock, and 10 (4.9%) had skin and soft tissue infections. Hypoactive delirium occurred in 84 (41%) of these patients, and the baseline characteristics of the patients are shown in Table 1. Hypertension, diabetes mellitus, APACHE II score ≥ 15, procalcitonin, CRP, IL-6 and lactate dehydrogenase had significant effects on the development of hypoactive delirium compared with the no delirium group (p < 0.05). Age, sex, chronic obstructive pulmonary disease (COPD), peripheral blood neutrophil count, lymphocyte count, hemoglobin, albumin, BNP, and magnesium were not significantly associated with hypoactive delirium (p > 0.05).

**Table 1:** General information of patients in the study.

Variable	Hypoactive delirium (n = 84)	No hypoactive delirium (n = 121)	*P* value
Age, median (IQR)	71 (59–78)	66 (56–78)	0.313
Male (n, %)	57 (67.9%)	69 (57%)	0.156
Hypertension (n, %)	46 (54.8%)	49 (40.5%)	0.031
COPD (n, %)	39(46.4%)	78 (64.5%)	0.341
Diabetes mellitus (n, %)	40 (47.6%)	32 (26.4%)	0.002
APACHE II score ≥ 15 (n, %)	65 (77.4%)	19 (15.7%)	< 0.001
Neutrophil, median (IQR)	6.06 (3.74–9.6)	9.69 (6.41–13.48)	0.111
Lymphocyte, median (IQR)	0.74 (0.45–1.05)	0.67 (0.42–1)	0.503
Hemoglobin, median (IQR)	128 (114–141)	128.5 (104.2–143.5)	0.648
Procalcitonin, median (IQR)	0.12 (0.04–0.47)	2.0 (0.04–8.44)	< 0.001
Albumin, median (IQR)	29.7 (26.1–33.5)	28.45 (25.95–31.33)	0.423
BNP, median (IQR)	724 (182.5–3150)	1152 (516–4673)	0.073
CRP, median (IQR)	21 (9.7–50.94)	91.95 (27.32–185.54)	< 0.001
LDH, median (IQR)	298 (217–356)	346 (238–495)	0.002
Magnesium, median (IQR)	0.9 (0.81–0.98)	0.9 (0.82–0.99)	0.416
Sodium, median (IQR)	137 (134–142)	138 (134–145)	0.429
IL-6, median (IQR)	17.85 (5.18–39.91)	156.08 (94.36–305.3)	< 0.001

APACHE II, Acute Physiology and Chronic Health Evaluation II; COPD, chronic obstructive pulmonary disease; BNP, brain natriuretic peptide; CRP, C-reactive protein; IL-6, interleukin-6; LDH, lactic dehydrogenase.

### Prediction risk factors by logistic regression analysis

To assess the predictive performance of the nomogram model, all risk measures were subjected to univariate and multivariate logistic regression models. A total of 17 variables, including sex, age, and hypertension status, were assessed. Three independent risk factors for hypoactive delirium were identified: diabetes mellitus (OR 3.305, 95% CI: 1.866–12.616; p = 0.047), CRP level (OR 1.002, 95% CI: 1.001–1.023; p = 0.044), and IL-6 level (OR 1.045, 95% CI: 1.017–1.063; p = 0.001) (Table 2). Odds ratios (ORs) were calculated and considered to be statistically significant if the 95% confidence interval (CI) was greater than 1.

**Table 2:** Multivariable analysis of risk factors related to hypoactive delirium.

Variables	β	OR	(95% CI)	*p* value
Constant	− 2.201	0.006		0.019
Diabetes mellitus	1.452	3.305	(1.866–12.616)	0.047
CRP	0.008	1.002	(1.001–1.023)	0.044
IL-6	0.044	1.045	(1.017–1.063)	0.001

OR, odds ratio; CI, confidence interval.

### Nomogram plot

**Figure 1:**
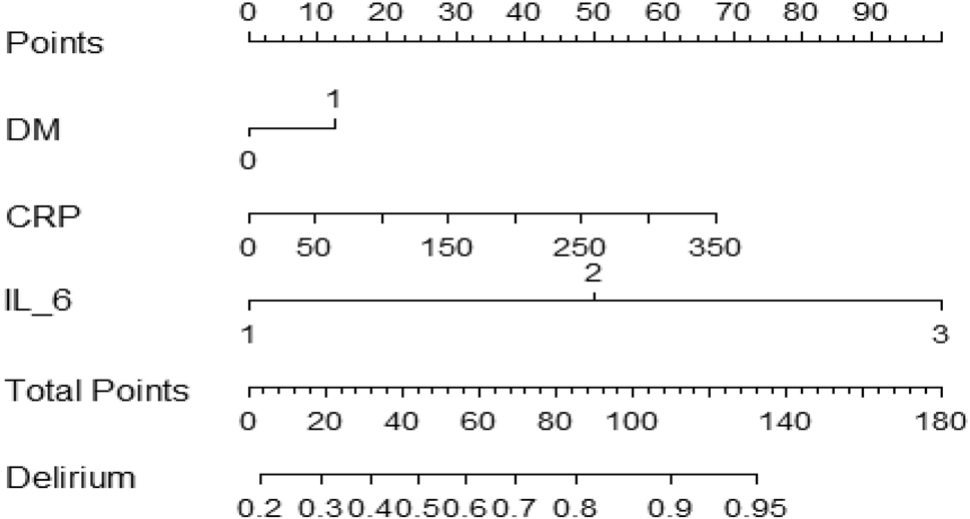
Nomogram used to predict the likelihood of hypoactive delirium in admitted ARDS patients. DM, diabetes mellitus; CRP, C-reactive protein; IL-6, interleukin 6.

A clinical prognostic model was developed to assess the likelihood of hypoactive delirium using three independent risk factors. The output of the model was generated in the form of a nomogram (Fig. 1). The likelihood of delirium was estimated by calculating the total score for the presence or absence of diabetes, CRP values, and IL-6 severity at admission. Using nomograms, we quantitatively analyzed whether each patient was predicted to develop hypoactive delirium.

### Identity and calibration

**Figure 2:**
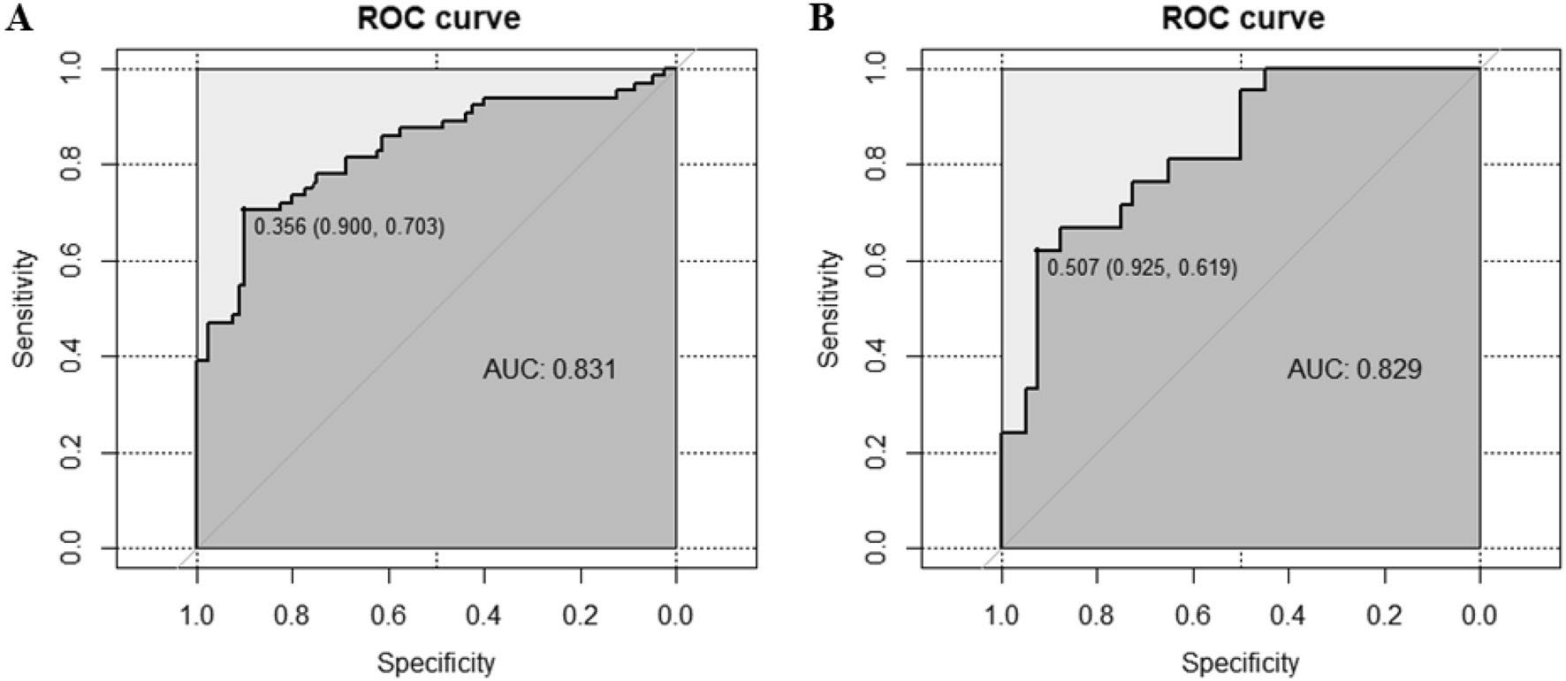
ROC curves for prognostic models in the (**A**) training set and (**B**) validation set. ROC: receiver operating characteristic; AUC: area under the curve.

**Figure 3:**
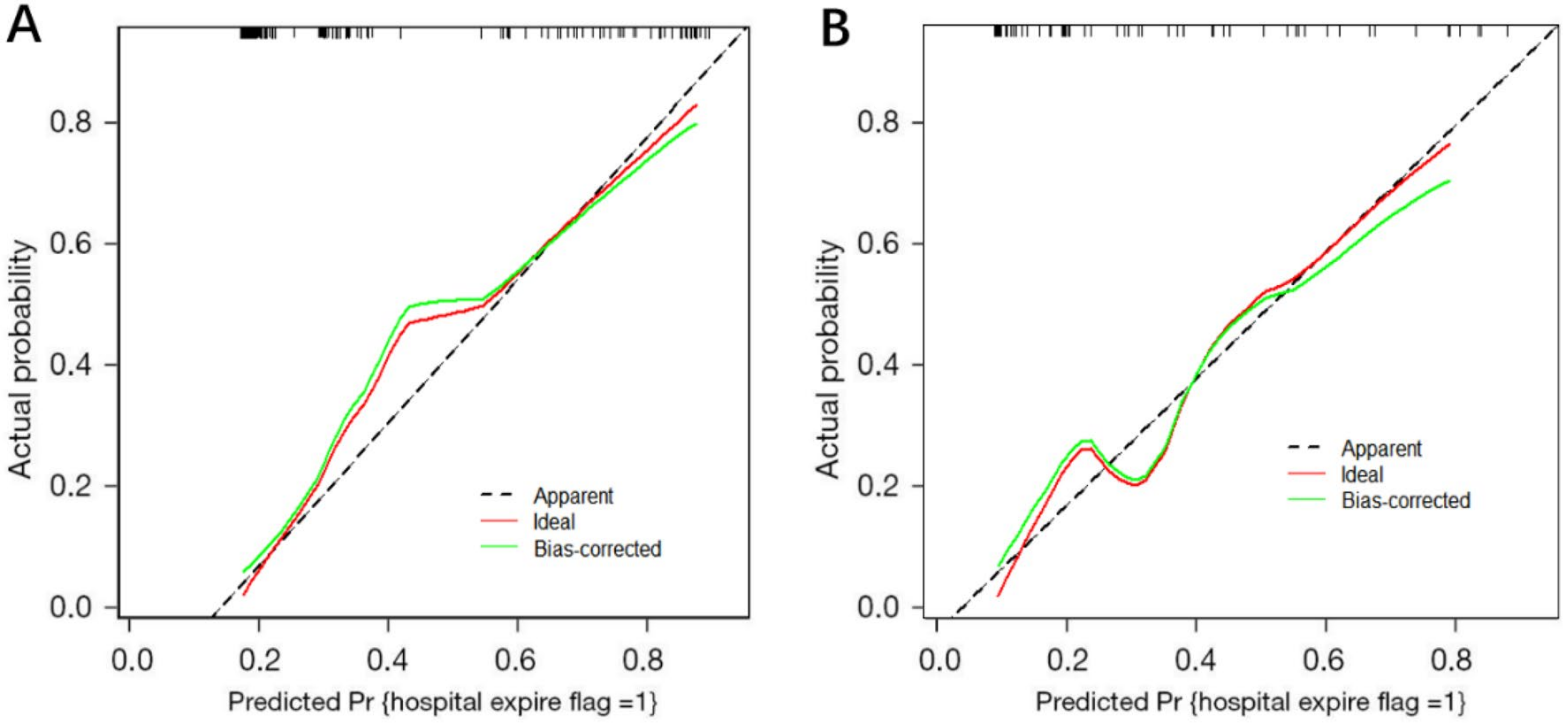
Calibration curve for the prognostic model. (**A**) Training set; (**B**) validation set.

To verify the universality, sensitivity and specificity of the above prognostic model for clinical application, we plotted receiver operating characteristic (ROC) curves (Fig. 2) and calibration curves (Fig. 3). The AUCs for the prognostic model were 0.831 and 0.829 in the training and validation sets, respectively. The cutoff value, specificity, and sensitivity of the prognostic model in the training sets were 0.356, 0.900, and 0.703, respectively, and those in the validation sets were 0.507, 0.925, and 0.619, respectively. This prognostic model showed high applicability in assessing hypoactive delirium outcomes in ARDS patients. In addition, the prognostic model was calibrated and analyzed using the bootstrap method with 1000 iterations. According to the calibration curve, the training and validation sets deviated slightly from the ideal curve but still maintained high accuracy.

### Decision curve analysis

**Figure 4:**
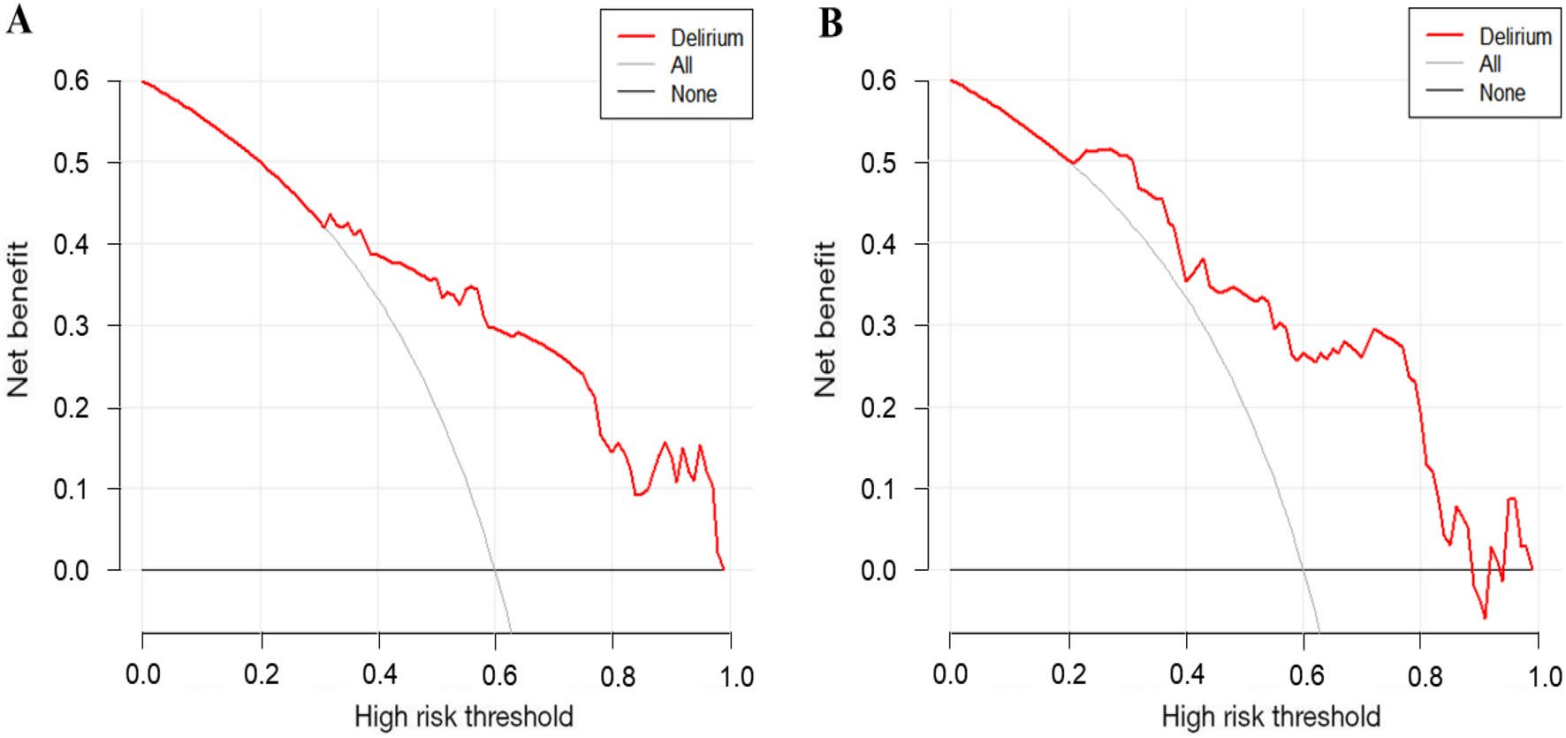
DCA curves for prognostic models. (**A**) Training set; (**B**) validation set.

To further assess the utility of the prognostic models in clinical practice, decision curve analysis was performed (Fig. 4). In the training set, the red curves indicate the accuracy of the prognostic model in predicting delirium outcome; gray vertical lines represent all patients with hypoactive delirium, and black horizontal lines represent no patients with hypoactive delirium. When the threshold probability (PT) in the training set was > 0.3, diabetes mellitus, CRP, and IL-6 in the prognostic model were highly beneficial as important indicators for predicting the occurrence of hypoactive delirium in ARDS patients and could be used to accurately assess whether patients developed hypoactive delirium. In the validation set, a nomogram-guided prognostic model efficiently distinguished the possibility of developing hypoactive delirium in patients when the PT was between 0.2 and 0.9.

## Discussion

A previous study reported that ARDS is an independent risk factor for the development of delirium in the ICU, and another study revealed that almost 100% of ARDS survivors experienced brain injury. More seriously, a fiftieth of ARDS patients die of severe neurological impairment^10^. In our study, 41% of ARDS patients presented with hypoactive delirium within 24 h of admission, which was a similar incidence to that reported in previous studies^8^. Our study revealed that hypoactive delirium is a predominant subtype of delirium in ARDS patients without trauma. The clinical symptoms of hypoactive delirium are characterized by drowsiness, apathy, and little speech, giving physicians the impression of a stable condition^9,11^. Therefore, early, effective and rapid detection of delirium is particularly important.

The CAM-ICU is one of the most common tools for diagnosing ICU delirium, but the examination process is time-consuming and labor-intensive. Moreover, face-to-face inquiry easily causes unpleasant experiences such as mental stress and anxiety in patients with no delirium^15^. Therefore, developing rapid and accurate recognition tools/models is particularly important for the early detection of hypoactive delirium. In our study, we found that an APACHE II score ≥ 15; diabetes mellitus; and high procalcitonin, lactic dehydrogenase, BNP, CRP and plasma IL-6 levels were significant variables in the univariate analysis (p < 0.05). We determined that diabetes mellitus (OR = 3.305), high CRP (OR = 1.002), and high plasma IL-6 (OR = 1.045) were independent risk factors for ARDS-associated hypoactive delirium.

Numerous studies have reported that diabetes mellitus is closely related to the development of delirium. Kirresh et al.^16^ reported a case of hypoactive delirium induced by hypoglycemic hemiparesis, and Kotfis et al.^17^ reported that diabetes and elevated HbA1c levels are risk factors for delirium after cardiac surgery. Some systematic reviews and meta-analyses also revealed that the incidence of delirium is significantly increased in diabetic patients^18,19^. One theory is that advanced glycation end products disrupt the normal function of the blood‒brain barrier and that controlling blood glucose can effectively reduce neuroinflammation^20^. Another theory for the development of delirium is decreased activity of the brain insulin signaling system, mainly due to central insulin resistance and insulin deficiency. Intranasally administered insulin (INI) can restore the activity of the brain insulin system and has wide therapeutic potential for treatment of delirium^21^. In 2022, a retrospective cohort study of 1404 subjects revealed that metformin reduced the incidence and mortality of delirium in patients with diabetes mellitus^22^.

Our data on the relationship between CRP and hypoactive delirium are similar to previous results. High levels of CRP have been associated with a high incidence, longer duration, and greater severity of delirium^23,24^. However, the relationship between CRP and delirium mortality remains unclear. One hypothesis suggests that inflammation can lead to endothelial cell and blood–brain barrier (BBB) dysfunction and induce local microbleeding or microthrombosis, further leading to brain injury^10^. Effective control of infection and a reduction in the inflammatory response may become treatments for hypoactive delirium.

Our study showed that higher blood IL-6 levels were associated with a greater incidence of hypoactive delirium, consistent with the findings of other researchers. Huang et al.^26^ and Chen et al.^25^ found that IL-6 was an independent risk factor for postoperative delirium. An increasing amount of evidence suggests that high systemic plasma IL-6, which can disrupt the integrity of the blood–brain barrier (BBB), increase solute permeability, and alter brain metabolism, directly causes neuronal apoptosis^10^. Experimental studies have also reported that peripheral plasma IL-6 causes a large number of biochemical factors to enter the brain, activate microglia, and release proinflammatory factors, leading to neuroinflammation^27,28^. Two experimental studies demonstrated that systemic anti-IL-6 therapy can effectively relieve delirium^28,29^. Another experimental study suggested that Yokukansan and glycyrrhizic acid can be used to treat hypoactive delirium by decreasing IL-6 levels in the hippocampus^30^. To date, there is no known drug or nondrug treatment for hypoactive delirium. Systemic anti-IL-6 agents may be potential therapeutic options in the future. One limitation of our study was that it was a single-center study. Future collaboration with multiple centers to increase the sample size could validate the accuracy and specificity of this model.

In summary, our results suggest that these prediction models are helpful for rapid diagnosis of delirium. The independent risk factors related to delirium may provide therapeutic targets for the treatment of delirium in the future. Metformin and interleukin-6 antagonists may become effective anti-delirium drugs.

## Conclusion

Hypoactive delirium is a common serious complication in nontraumatic ARDS patients. To date, the pathophysiological mechanism of hypoactive delirium is unclear, and treatment methods are lacking. Our logistic regression model not only effectively predicts hypoactive delirium in a timely manner but also reveals potential clinical therapeutic targets. Notably, IL-6 inhibitors are expected to be therapeutic agents in the future.

## Data Availability

The authors affirm that all the data are true and valid. The datasets used and/or analyzed during the current study are available from the corresponding author upon reasonable request.

## Abbreviations

APACHE II: Acute Physiology and Chronic Health Evaluation II
ARDS: Acute respiratory distress syndrome
ICU: Intensive care unit
CRP: C-reactive protein
IL-6: Interleukin-6
ROC: Receiver operating characteristic
DCA: Decision curve analysis
CAM-ICU: Confusion Assessment Method for the Intensive Care Unit
RASS: Richmond Agitation-Sedation Scale
BNP: Brain natriuretic peptide
HIS: Hospital information system
AUC: Area under the curve
OR: Odds ratio
CI: Confidence interval
PT: Threshold probability
BBB: Blood–brain barrier
